# Joint Learning of Correlation-Constrained Fuzzy Clustering and Discriminative Non-Negative Representation for Hyperspectral Band Selection

**DOI:** 10.3390/s23104838

**Published:** 2023-05-17

**Authors:** Zelin Li, Wenhong Wang

**Affiliations:** College of Computer Science, Liaocheng University, Liaocheng 252059, China

**Keywords:** hyperspectral band selection, constrained fuzzy C-means, graph regularized non-negative matrix factorization, non-negative representation, alternating direction multiplier method

## Abstract

Hyperspectral band selection plays an important role in overcoming the curse of dimensionality. Recently, clustering-based band selection methods have shown promise in the selection of informative and representative bands from hyperspectral images (HSIs). However, most existing clustering-based band selection methods involve the clustering of original HSIs, limiting their performance because of the high dimensionality of hyperspectral bands. To tackle this problem, a novel hyperspectral band selection method termed joint learning of correlation-constrained fuzzy clustering and discriminative non-negative representation for hyperspectral band selection (CFNR) is presented. In CFNR, graph regularized non-negative matrix factorization (GNMF) and constrained fuzzy C-means (FCM) are integrated into a unified model to perform clustering on the learned feature representation of bands rather than on the original high-dimensional data. Specifically, the proposed CFNR aims to learn the discriminative non-negative representation of each band for clustering by introducing GNMF into the model of the constrained FCM and making full use of the intrinsic manifold structure of HSIs. Moreover, based on the band correlation property of HSIs, a correlation constraint, which enforces the similarity of clustering results between neighboring bands, is imposed on the membership matrix of FCM in the CFNR model to obtain clustering results that meet the needs of band selection. The alternating direction multiplier method is adopted to solve the joint optimization model. Compared with existing methods, CFNR can obtain a more informative and representative band subset, thus can improve the reliability of hyperspectral image classifications. Experimental results on five real hyperspectral datasets demonstrate that CFNR can achieve superior performance compared with several state-of-the-art methods.

## 1. Introduction

Hyperspectral images (HSIs) are typically generated by capturing hundreds of narrow and continuous electromagnetic bands from the radiation of ground objects via hyperspectral sensors. Thus, HSIs can provide an abundance of spectral and spatial information regarding target objects [1]. HSIs are currently used in a wide variety of applications, such as target detection [2], land cover classification [3], urban management [4], and soil investigation [5]. Hyperspectral classification is an important task in such applications because it can identify the category of the existing land cover in each pixel of an HSI. However, HSIs usually have high-dimensional features and large amounts of redundant information, inducing the Hughes phenomenon in the applications of hyperspectral classification [6]. Band selection that reduces redundant information by selecting a set of representative bands from an HSI is an effective method to tackle this problem [7].

Band selection methods are generally categorized into two categories: supervised and unsupervised methods. Supervised methods rely on labeled samples, which are typically costly to obtain in practice [8]. Conversely, unsupervised methods can perform band selection without using labeled samples, providing greater flexibility in practical applications [9]. Over the past few decades, researchers have introduced various unsupervised methods, which can be categorized as ranking, searching, and clustering-based methods. Ranking-based methods commonly use certain indicators to measure the significance of each band, such as maximum variance-based principal component analysis (MVPCA) [10] and density-based spatial clustering of applications with noise [11]. Unfortunately, the performance of ranking-based methods is limited because they rarely consider the high correlation between bands [12]. Searching-based methods usually select the representative bands based on effective objective functions used to optimize given criteria, such as the volume gradient-based band selection method [13] and particle swarm optimization-based method [14]. However, these methods have high computational complexity during optimization [4]. Clustering-based methods first perform clustering on all bands, and then select a representative band from each cluster. Typical examples include Ward’s linkage strategy using divergence (WaLuDi) [15], enhanced fast density-peak-based clustering (E-FDPC) [16], adaptive subspace partition strategy (ASPS) [9], and region-aware hierarchical latent feature representation learning-guided clustering (HLFC) [17].

Clustering-based methods can generally provide superior performance by considering the similarity among bands [16]. However, the performance of clustering-based methods is degraded when handling high-dimensional data [17]. The concept of combining representation learning with clustering has been applied to many fields to improve clustering performance in the case of high-dimensional data. For example, He et al. [18] proposed a spatial weighted matrix distance-based fuzzy clustering algorithm that first uses variable-based principal component analysis for dimensionality reduction, and then divides multivariate time series data into different clusters. Gu et al. [19] introduced fuzzy double C-means based on the sparse self-representation method, in which a discriminative feature set is obtained via sparse self-representation followed by the use of the fuzzy double C-means to obtain superior clustering results. Notably, the aforementioned studies reveal that representation learning and clustering remain independent stages. Some researchers have proposed integrating representation learning and clustering into one framework. For instance, Lu et al. [20] presented subspace clustering constrained sparse non-negative matrix factorization (SC-NMF) for hyperspectral unmixing. In SC-NMF, subspace clustering is embedded into the non-negative matrix factorization to extract endmembers and corresponding abundance accurately. The joint framework of representation learning and clustering has yielded excellent results in some practical applications but has not been investigated in band selection applications. Therefore, it is a challenge to design an effective joint model of clustering and representative learning for the band selection task and introduce appropriate regularizations into the joint model based on problem-dependent information.

To address the abovementioned issues, a novel clustering-based band selection method called joint learning of correlation-constrained fuzzy clustering and discriminative non-negative representation (CFNR) is proposed in this paper. CFNR aims to perform clustering on the discriminative non-negative representation of all bands rather than on the original high-dimensional hyperspectral bands, as well as fully consider the band correlation property and intrinsic manifold structure of HSIs, by which an informative and representative band subset for hyperspectral classification is selected. Specifically, CFNR can perform clustering and representation learning jointly on the target HSIs by integrating the objective function of graph regularized non-negative matrix factorization (GNMF) into the constrained fuzzy C-means (FCM) model. Therefore, effective clustering results are expected to be obtained by conducting fuzzy clustering on the feature representation of bands learned by GNMF. Specifically, GNMF is used in CFNR to obtain discriminative non-negative representation of all bands by taking advantage of the intrinsic manifold structure of HSIs. Furthermore, a correlation constraint is imposed on the FCM membership matrix to exploit prior information regarding the existing strong correlation between neighboring bands in an HSI, by which adjacent bands are enforced to possess similar cluster assignments. This condition is expected to improve the effectiveness of clustering for band selection. Finally, an information entropy-based method is employed to select a representative band from each cluster. Five real hyperspectral datasets are used to evaluate the performance of CFNR and compare it with five representative methods, demonstrating that the proposed CFNR method can provide superior performance. The main contributions of this study are listed as follows.

- A novel band selection method called CFNR, by which fuzzy clustering and learning of discriminative non-negative representation can be performed simultaneously, is developed to select representative bands for the classification task of HSIs. CFNR conducts clustering on the discriminative non-negative representation of all bands rather than the original high-dimensional hyperspectral bands. Compared with existing related band selection methods, the advantage of the proposed CFNR is the integration of representation learning and clustering into a unified model.
- A correlation constraint based on problem-dependent information is imposed on the membership matrix in the CFNR model to take full advantage of the high correlation between adjacent bands in HSIs. Furthermore, GNMF, through which the intrinsic manifold structure of HSIs can be fully exploited, is utilized in CFNR to learn the discriminative non-negative representation of all bands. In addition, the alternating direction multiplier method (ADMM) is used to solve the optimization problem furnished by the proposed joint model.
- The performance of our proposed method is validated through comparison with five representative band selection methods on five real datasets. Experimental results show that CFNR demonstrates superior performance for band selection.

The remainder of this paper is organized as follows. Section 2 introduces the theoretical foundations of the research problem. Section 3 presents the proposed model as well as the solution. In Section 4, the, details of the experimental results from the five real datasets and the corresponding analysis are presented, including a discussion of the findings from our experiments, advantages, and limitations of our method. Finally, the paper is summarized in Section 5.

## 2. Related Work

### 2.1. Constrained FCM

FCM, one of the most popular fuzzy clustering methods, divides a dataset $X=\left[x_{1},x_{2},\ldots ,x_{L}\right]\in R^{N\times L}$ into *K* clusters using a soft clustering assignment strategy. Compared with hard clustering methods, such as K-means, the advantage of FCM lies in its use of a membership matrix $U\in R^{K\times L}$ to indicate the assignment probability of each sample to different clusters [21]. For example, $U_{k,l}$ is used to represent the probability that data point $X_{:,l}$ belongs to the *k*-th cluster. FCM can be formally expressed as a minimization problem as follows.

(1) $$\begin{array}{c} \min_{U,C}\sum_{k=1}^{K}\sum_{l=1}^{L}U_{k,l}^{m}||{\left(X^{T}\right)}_{l,:}-C_{k,:}|{|}^{2},\;s.t.\;\sum_{k=1}^{K}U_{k,l}=1, \end{array}$$

where $C=[c_{1},c_{2},\ldots ,c_{k},\ldots ,c_{K}]$ denotes a matrix formed by the centroid vector $c_{k}$, k=1,2,…,K; m>1 denotes the fuzzification factor and is set to 2 in this study; ||·|| denotes the Euclidean distance between the *l*-th data point and *k*-th cluster center.

The objective function of problem (1) is nonconvex; thus, the solutions of FCM are prone to trapping into local minima and are sensitive to the initial values [22]. To better address the needs of a specific application, different constraints are imposed on solution U in problem (1) [23,24]. Thus, the general model of the constraint-based FCM can be given as

(2) $$\begin{array}{c} \min_{U,C}\sum_{k=1}^{K}\sum_{l=1}^{L}U_{k,l}^{m}||{\left(X^{T}\right)}_{l,:}-C_{k,:}|{|}^{2}+\beta g\left(U\right),\;s.t.\;\sum_{k=1}^{K}U_{k,l}=1, \end{array}$$

where g(U) denotes the regularization term used to impose the specific constraint on U and β represents the corresponding regularization parameter.

### 2.2. GNMF

Non-negative matrix factorization (NMF) has become a widely used method for low-dimensional representation learning of high-dimensional data owing to its simple structure and meaningful explainability [25]. Numerous variants of NMF have been recently proposed to further improve NMF performance in different applications [26,27]. For example, GNMF [28], which introduces graph-based regularization into the standard NMF, was proposed to exploit the intrinsic manifold structure of data. Rather than the standard NMF, GNMF is used in the current study to achieve the discriminative non-negative representation of all bands in an HSI. Specifically, NMF aims to find two low-rank non-negative matrices $A\in R^{N\times P}$ and $S\in R^{L\times P}$ that satisfy the condition $X\approx AS^{T}$ [29]. The NMF model can generally be given as

(3) $$\begin{array}{c} \min_{A,S}||X-AS^{T}|{|}_{F}^{2},\;s.t.\;A,S\geq 0, \end{array}$$

where $||\cdot |{|}_{F}$ represents the Frobenius norm of matrices and A and S represent the basis and coefficient matrices, respectively. The GNMF model [28] is defined on the basis of the standard NMF model by first constructing a graph indicating the K-neighbors of each sample and then adding a regularization term based on the obtained graph. The GNMF model can formally be expressed as

(4) $$\begin{array}{c} \min_{A,S}||X-AS^{T}|{|}_{F}^{2}+\frac{\lambda }{2}\mathrm{Tr}\left(S^{T}LS\right),\;s.t.\;A,S\geq 0, \end{array}$$

with

(5) $$\begin{array}{c} L=D-W, \end{array}$$

where Tr(·) denotes the trace of the corresponding matrices; L represents the graph Laplacian matrix; W represents the weight matrix of the graph; and D represents a diagonal matrix with $D_{ii}=\sum_{j}W_{ij}$.

## 3. Proposed Methodology

**Figure 1:**
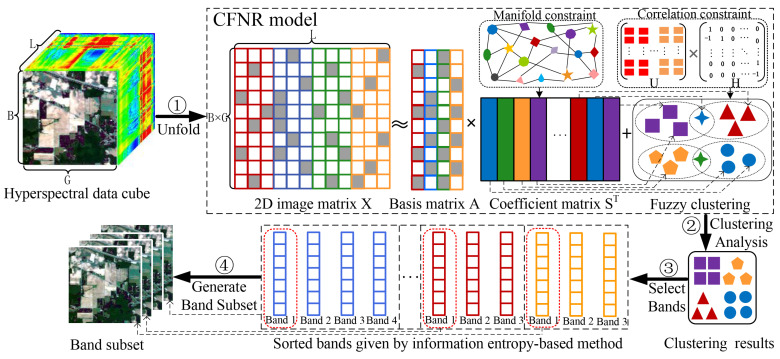
Flowchart of the proposed CFNR method.

Figure 1 illustrates a flowchart of the proposed method, in which clustering assignments and representation learning are jointly achieved. Specifically, the original hyperspectral data cube is first transformed into a two-dimensional matrix representation. Subsequently, GNMF is applied to learn a low-dimensional non-negative representation of the HSIs with clustering discriminability by exploiting the intrinsic manifold structure of HSIs. Furthermore, correlation-constrained FCM, which can effectively preserve the local similarity between the membership vectors of adjacent bands, is adopted on the basis of the obtained feature representation of each band to conduct clustering analysis for all the bands. Representative bands for subsequent classification tasks are then generated by selecting a band from each cluster using an information entropy-based method.

### 3.1. Correlation-Constrained FCM

To make full use of the high correlation among adjacent bands during clustering, we design an efficient correlation constraint for FCM. This constraint is designed on the principle that similar samples should have similar cluster assignments. Specifically, the design of the correlation constraint is inspired by the total variation (TV) regularization [30], which has been demonstrated to be quite efficient for image recovery and imaging inverse problems [31]. According to [32], TV regularization could be expressed as $TV\left(S\right)=\sum_{j\in NS(i)}||s_{i}-s_{j}|{|}_{1}$, where $S=[s_{1},s_{2},\ldots ,s_{n},\ldots ,s_{N}]$ indicates the matrix formed by the pixel $s_{n}$, n=1,2,…,N, NS(i) denotes the set of indexes of neighboring pixels of the *i*-th pixel, and $||\cdot |{|}_{1}$ represents the l1-norm of vectors. For the convenience of computation, the TV regularization can be implemented by the matrix operation $||FS|{|}_{1,1}$, where $||S|{|}_{1,1}=\sum_{i=1}^{M}||S_{i}|{|}_{1}$ and F denotes the linear operator used to compute the differences between $s_{i}$ and its neighbors. In this study, the correlation constraint is imposed on matrix U via the regularization term g(U), which is given by

(6) $$g\left(U\right)=||\mathrm{UH}|{|}_{1,1},$$

where linear operator $H\in R^{L\times (L-1)}$ is used to compute the difference between two adjacent bands. Specifically, the difference vector $d_{l}$, l=1,2,…,(L−1), which denotes the difference between bands $X_{:,l}$ and $X_{:,(l+1)}$ are computed using $UH=[d_{1},d_{2},\ldots ,d_{l},\ldots ,d_{\left(L-1\right)}]$ with H defined by

(7) $$H=\left[\begin{array}{ccccc} 1\; & 0\; & 0 & \cdots & 0\\ -1\; & 1\; & 0 & \cdots & 0\\ 0\; & -1\; & 1 & \cdots & 0\\ 0\; & 0\; & -1 & \cdots & 0\\ \vdots \; & \vdots \; & \vdots & \ddots & \vdots \;\\ 0\; & 0\; & 0 & \cdots & 1\;\\ 0\; & 0\; & 0 & \cdots & -1\; \end{array}\right].$$

Based on the abovementioned definition, the correlation-constrained FCM can be written as

(8) $$\begin{array}{cc} \min_{U,C}\sum_{k=1}^{K}\sum_{l=1}^{L} & U_{k,l}^{m}||{\left(X^{T}\right)}_{l,:}-C_{k,:}|{|}^{2}+\beta ||\mathrm{UH}|{|}_{1,1},\;s.t.\;\sum_{k=1}^{K}U_{k,l}=1. \end{array}$$

### 3.2. CFNR Model

Traditional clustering-based band selection methods typically fail to provide good clustering results owing to the high dimensionality of the original hyperspectral bands. To address this problem, discriminative non-negative representation learning is applied to each band of the target HSI. This is inspired by a study [33] in which hyperspectral bands were expressed as sparse linear representations of several basis vectors via NMF [33]. In this study, the objective function of GNMF in Equation (4) is introduced into the model of correlation-constrained FCM in Equation (8) to simultaneously perform non-negative representation learning and clustering. Consequently, the CFNR model can be expressed as

(9) $$\begin{array}{cc} \min_{U,C,A,S}\sum_{k=1}^{K}\sum_{l=1}^{L}U_{k,l}^{m}||S_{l,:}-C_{k,:}|{|}^{2}+\frac{\alpha }{2}||X-AS^{T}|{|}_{F}^{2}+\; & \frac{\lambda }{2}\mathrm{Tr}\left(S^{T}LS\right)+\beta ||UH|{|}_{1,1},\\ & s.t.\;\sum_{k=1}^{K}U_{k,l}^{m}=1,A\geq 0,S\geq 0. \end{array}$$

Overall, the CFNR model demonstrates the following advantages.

1. CFNR combines non-negative representation learning and clustering into one model to improve the performance of band selection.
2. CFNR can learn discriminative non-negative representation based on manifold learning by preserving the internal manifold structure of HSIs in a low-dimensional space.
3. CFNR maximizes the strong correlation between adjacent bands of HSIs, which is beneficial for obtaining superior clustering results for band selection.

### 3.3. Solution of the CFNR Model

ADMM [34] is an effective method for solving large optimization problems. The principle of ADMM is to break up the problem into subproblems that can be solved iteratively by introducing additional variables [35]. Alternate optimization is achieved by fixing other variables and optimizing the desired variables [36]. In this study, ADMM is adopted to solve the optimization problem expressed in Equation (9). To facilitate model optimization, the non-negative constraint is first integrated into the objective function in Equation (9), which can be rewritten as

(10) $$\begin{array}{cc} \min_{U,C,A,S}\sum_{k=1}^{K}\sum_{l=1}^{L}U_{k,l}^{m}||S_{l,:}-C_{k,:}|{|}^{2}\; & +\frac{\alpha }{2}||X-AS^{T}|{|}_{F}^{2}+\frac{\lambda }{2}\mathrm{Tr}\left(S^{T}LS\right)+\beta ||UH|{|}_{1,1}\\ & \;\;{+}_{lR+}\left(A\right){+}_{lR+}\left(S\right),\;s.t.\sum_{k=1}^{K}U_{k,l}=1, \end{array}$$

where ${}_{lR+}\left(A\right)$ is an indicator function that has the value of zero if each entry of matrix A is non-negative; otherwise it has the value +∞.

To solve the optimization problem given in Equation (10) via ADMM, seven auxiliary variables, $V_{1},V_{2},V_{3},V_{4},V_{5},V_{6}$, and $V_{7}$ are introduced into the objective function in Equation (10). Subsequently, the optimization problem is reformulated as

(11) $$\begin{array}{cc} \; & \min_{\begin{array}{c} U,C,A,S,V_{1},V_{2},\\ V_{3},V_{4},V_{5},V_{6},V_{7} \end{array}}\sum_{k=1}^{K}\sum_{l=1}^{L}U_{k,l}^{m}||{\left(V_{1}\right)}_{l,:}-C_{k,:}|{|}^{2}+\frac{\alpha }{2}||X-AV_{2}^{T}|{|}_{F}^{2}+\frac{\lambda }{2}\mathrm{Tr}\left(V_{5}^{T}LV_{5}\right)+\beta ||V_{4}|{|}_{1,1}\\ & \;\;\;{+}_{lR+}\left(V_{7}\right){+}_{lR+}\left(V_{6}\right),\\ & s.t.\sum_{k=1}^{K}U_{k,l}=1,V_{1}=S,V_{2}=S,V_{3}=U,V_{4}=V_{3}H,V_{5}=S,V_{6}=S,V_{7}=A. \end{array}$$

Based on the objective function in Equation (11), the augmented Lagrange function is written as

(12) $$\begin{array}{cc} \; & L(U,C,A,S,V_{1},V_{2},V_{3},V_{4},V_{5},V_{6},V_{7},Z_{1},Z_{2},Z_{3},Z_{4},Z_{5},Z_{6},Z_{7})\\ \; & =\sum_{k=1}^{K}\sum_{l=1}^{L}U_{k,l}^{m}||{\left(V_{1}\right)}_{l,:}-C_{k,:}|{|}^{2}+\frac{\alpha }{2}||X-AV_{2}^{T}|{|}_{F}^{2}+\frac{\lambda }{2}\mathrm{Tr}\left(V_{5}^{T}LV_{5}\right)+\beta ||V_{4}|{|}_{1,1}{+}_{lR+}\left(V_{6}\right)\\ \; & {+}_{lR+}\left(V_{7}\right)-\langle Z_{7},A-V_{7}\rangle +\frac{\rho }{2}||A-V_{7}|{|}_{F}^{2}-\langle Z_{1},S-V_{1}\rangle +\frac{\rho }{2}||S-V_{1}|{|}_{F}^{2}-\langle Z_{2},S-V_{2}\rangle \\ \; & +\frac{\rho }{2}||S-V_{2}|{|}_{F}^{2}-\langle Z_{3},U-V_{3}\rangle +\frac{\rho }{2}||U-V_{3}|{|}_{F}^{2}-\langle Z_{4},V_{3}H-V_{4}\rangle +\frac{\rho }{2}||V_{3}H-V_{4}|{|}_{F}^{2}\\ \; & -\langle Z_{5},S-V_{5}\rangle +\frac{\rho }{2}||S-V_{5}|{|}_{F}^{2}-\langle Z_{6},S-V_{6}\rangle +\frac{\rho }{2}||S-V_{6}|{|}_{F}^{2}, \end{array}$$

where matrices $Z_{1}$, $Z_{2}$, $Z_{3}$, $Z_{4}$, $Z_{5}$, $Z_{6}$, and $Z_{7}$ are Lagrange multipliers; ρ > 0 is the penalty parameter; and 〈·〉 denotes the inner product operator.

Next, we apply ADMM to optimize variables A, S, U, C, $V_{1}$, $V_{2}$, $V_{3}$, $V_{4}$, $V_{5}$, $V_{6}$, and $V_{7}$ according to Equation (12). Note that *t* denotes the number of iterations.

**A**-update: To perform an optimization of A, we ignore terms that are not related to A in the objective function given in Equation (12). The simplified optimization problem can be given as

(13) $$\begin{array}{cc} A^{\left(t+1\right)} & =\underset{A}{\arg\min}\frac{\alpha }{2}||X^{t}-A^{t}{\left(V_{2}^{T}\right)}^{t}|{|}_{F}^{2}+\frac{\rho }{2}||A^{t}-V_{7}^{t}-\zeta_{7}^{t}|{|}_{F}^{2}. \end{array}$$

By setting the derivative of the objective function in Equation (13) to zero, the solution of A can be obtained as

(14) $$\begin{array}{c} A^{\left(t+1\right)}=\left(\rho V_{7}^{t}+\rho \zeta_{7}^{t}+\alpha X^{t}V_{2}^{t}\right){\left(\alpha {\left(V_{2}^{T}\right)}^{t}V_{2}^{t}+\rho I\right)}^{\left(-1\right)}, \end{array}$$

where I denotes the identity matrix, $\zeta_{2}=Z_{2}/\rho$, and $\zeta_{7}=Z_{7}/\rho$.

**$V_{7}$**-update: The variable $V_{7}$ is optimized in accordance with Equation (15):

(15) $$\begin{array}{c} V_{7}^{\left(t+1\right)}=\underset{V_{7}}{\arg\min}{}_{lR+}\left(V_{7}^{t}\right)+\frac{\rho }{2}||A^{\left(t+1\right)}-V_{7}^{t}-\zeta_{7}^{t}|{|}_{F}^{2}. \end{array}$$

According to Equation (15), $A^{\left(t+1\right)}-\zeta_{7}^{t}$ needs to be projected onto the non-negative quadrant. This is achieved by the update rule of $V_{7}$ Equation (16):

(16) $$\begin{array}{c} V_{7}^{\left(t+1\right)}=\max\left(A^{\left(t+1\right)}-\zeta_{7}^{t},0\right). \end{array}$$

**C**-update: According to Equation (12), matrix C can be optimized by solving the sub-optimization problem given in Equation (17):

(17) $$\begin{array}{c} C_{k,:}^{\left(t+1\right)}=\underset{C}{\arg\min}\sum_{k=1}^{K}\sum_{l=1}^{L}{\left(U_{k,l}^{m}\right)}^{t}||{\left(V_{1}^{t}\right)}_{l,:}-C_{k,:}^{t}|{|}^{2}. \end{array}$$

Based on Equation (17), the optimization process of C is the same as that in the standard FCM. Thus, the update rule of C can be written as Equation (18):

(18) $$\begin{array}{c} C_{k,:}^{\left(t+1\right)}=\frac{\sum_{k=1}^{K}{\left(U_{k,l}^{m}\right)}^{t}{\left(V_{1}^{t}\right)}_{l,:}}{\sum_{k=1}^{K}{\left(U_{k,l}^{m}\right)}^{t}}. \end{array}$$

**S**-update: By fixing other variables that are not related to the variable S in Equation (12), the suboptimization problem Equation (19) about S can be written as

(19) $$\begin{array}{c} S^{\left(t+1\right)}=\underset{S}{\arg\min}\frac{\rho }{2}||S^{t}-V_{1}^{t}-\zeta_{1}^{t}|{|}_{F}^{2}+\frac{\rho }{2}||S^{t}-V_{2}^{t}-\zeta_{2}^{t}|{|}_{F}^{2}\\ \;+\frac{\rho }{2}||S^{t}-V_{5}^{t}-\zeta_{5}^{t}|{|}_{F}^{2}+\frac{\rho }{2}||S^{t}-V_{6}^{t}-\zeta_{6}^{t}|{|}_{F}^{2}. \end{array}$$

By setting the derivative of the objective function in Equation (19) to zero, the optimization of S can be simply expressed as

(20) $$\begin{array}{c} S^{\left(t+1\right)}=\frac{1}{4}\left(V_{1}^{t}+\zeta_{1}^{t}+V_{2}^{t}+\zeta_{2}^{t}+V_{5}^{t}+\zeta_{5}^{t}+V_{6}^{t}+\zeta_{6}^{t}\right), \end{array}$$

where $\zeta_{1}=Z_{1}/\rho$, $\zeta_{5}=Z_{5}/\rho$, and $\zeta_{6}=Z_{6}/\rho$.

**$V_{1}$**-update: The suboptimization of about $V_{1}$ can be expressed as Equation (21) by fixing irrelevant variables in Equation (12):

(21) $$\begin{array}{c} V_{1}^{\left(t+1\right)}=\underset{V_{1}}{\arg\min}\sum_{k=1}^{K}\sum_{l=1}^{L}{\left(U_{k,l}^{m}\right)}^{t}||{\left(V_{1}^{t}\right)}_{l,:}-C_{k,:}^{\left(t+1\right)}|{|}^{2}+\frac{\rho }{2}||V_{1}^{t}-S_{1}^{\left(t+1\right)}+\zeta_{1}^{t}|{|}_{F}^{2}\\ =\sum_{l=1}^{L}\sum_{k=1}^{K}{\left(U_{k,l}^{m}\right)}^{t}||{\left(V_{1}^{t}\right)}_{l,:}-C_{k,:}^{\left(t+1\right)}|{|}^{2}+\frac{\rho }{2}\sum_{l=1}^{L}||{\left(V_{1}^{t}\right)}_{l,:}-{\left(S_{1}^{\left(t+1\right)}\right)}_{l,:}+{\left(\zeta_{1}^{t}\right)}_{l,:}|{|}_{F}^{2}. \end{array}$$

We set the derivative of the objective function in Equation (21) to zero. Subsequently, the update rule of $V_{1}$ is obtained and formulated as

(22) $$\begin{array}{c} {\left(V_{1}^{\left(t+1\right)}\right)}_{l,:}=\frac{\sum_{k=1}^{K}{\left(U_{k,l}^{m}\right)}^{t}C_{k,:}^{t}+\frac{\rho }{2}\left({\left(S_{1}^{\left(t+1\right)}\right)}_{l,:}+{\left(\zeta_{1}^{t}\right)}_{l,:}\right)}{\sum_{k=1}^{K}{\left(U_{k,l}^{m}\right)}^{t}+\frac{\rho }{2}}. \end{array}$$

Using matrix operations, Equation (22) can be rewritten as

(23) $$\begin{array}{c} V_{1}^{\left(t+1\right)}={\left({\left(U^{m}\right)}^{t}\right)}^{T}C^{\left(t+1\right)}+\frac{\rho }{2}\left(S^{\left(t+1\right)}-\zeta_{1}^{t}\right))./{\left(\left(1*\sum {\left(U^{m}\right)}^{t}\right)\right)}^{T}+\frac{\rho }{2}), \end{array}$$

where 1 denotes a column vector of one.

**$V_{2}$**-update: Fixing variables that are irrelevant to $V_{2}$ in Equation (12), the subproblem concerning $V_{2}$ is formulated as

(24) $$\begin{array}{c} V_{2}^{\left(t+1\right)}=\underset{V_{2}}{\arg\min}\frac{\alpha }{2}||X-A^{\left(t+1\right)}{\left(V_{2}^{T}\right)}^{t}|{|}_{F}^{2}+\frac{\rho }{2}||V_{2}^{t}-S^{\left(t+1\right)}+{\zeta_{2}}^{t}|{|}_{F}^{2}. \end{array}$$

By setting the derivative of the objective function in Equation (24) to zero, the update rule of $V_{2}$ is expressed as

(25) $$\begin{array}{c} V_{2}^{\left(t+1\right)}=\left(\rho S^{\left(t+1\right)}-\rho {\zeta_{2}}^{t}+\alpha X^{T}A^{\left(t+1\right)}\right){\left(\alpha {\left(A^{T}\right)}^{\left(t+1\right)}A^{\left(t+1\right)}+\rho I\right)}^{\left(-1\right)}. \end{array}$$

**$V_{5}$**-update: To optimize $V_{5}$, the corresponding subproblem is formulated as Equation (26):

(26) $$\begin{array}{c} V_{5}^{\left(t+1\right)}=\underset{V_{5}}{\arg\min}\frac{\lambda }{2}\mathrm{Tr}\left({\left(V_{5}^{T}\right)}^{t}LV_{5}^{t}\right)+\frac{\rho }{2}||V_{5}^{t}-S^{\left(t+1\right)}+{\zeta_{5}}^{t}|{|}_{F}^{2}. \end{array}$$

We set the derivative of the objective function in Equation (26) to zero and obtain

(27) $$\begin{array}{c} V_{5}^{\left(t+1\right)}={\left(\lambda L+\rho I\right)}^{\left(-1\right)}\left(\rho S^{\left(t+1\right)}-\rho {\zeta_{5}}^{t}\right). \end{array}$$

**$V_{6}$** -update: The suboptimization problem regarding $V_{6}$ is shown by Equation (28):

(28) $$\begin{array}{c} V_{6}^{\left(t+1\right)}=\underset{V_{6}}{\arg\min}{}_{lR+}\left(V_{6}^{t}\right)+\frac{\rho }{2}||S^{\left(t+1\right)}-V_{6}^{t}-\zeta_{6}^{t}|{|}_{F}^{2}. \end{array}$$

The update rule of $V_{6}$ is obtained by projecting $S^{\left(t+1\right)}-\zeta_{6}^{t}$ onto the non-negative quadrant, expressed as

(29) $$\begin{array}{c} V_{6}^{\left(t+1\right)}=\max\left(S^{\left(t+1\right)}-\zeta_{6}^{t},0\right). \end{array}$$

**U**-update: We derive the update steps of U by referring to [35]. Specifically, the subproblem of U can be reformulated as Equation (30):

(30) $$\begin{array}{cc} U^{\left(t+1\right)} & =\underset{U}{\arg\min}\sum_{k=1}^{K}\sum_{l=1}^{L}{\left(U_{k,l}^{2}\right)}^{t}||{\left(V_{1}^{\left(t+1\right)}\right)}_{l,:}-C_{k,:}^{\left(t+1\right)}|{|}^{2}-\langle Z_{3}^{t},U^{t}-V_{3}^{t}\rangle \\ \; & \;+\frac{\rho }{2}||U^{t}-V_{3}^{t}|{|}_{F}^{2},\;\;s.t.\sum_{k=1}^{K}U_{k,l}=1. \end{array}$$

Subsequently, Equation (30) can be rewritten as

(31) $$\begin{array}{c} \underset{U}{\arg\min}\sum_{k=1}^{K}\sum_{l=1}^{L}\left({\tilde{A}}^{t}{\left(U_{k,l}^{2}\right)}^{t}+{\tilde{B}}^{t}U_{k,l}^{t}\right),\;s.t.\sum_{k=1}^{K}U_{k,l}=1, \end{array}$$

with

(32) $$\begin{array}{c} {\tilde{A}}^{t}=||{\left(V_{1}\right)}_{l,:}^{\left(t+1\right)}-C_{k,:}^{\left(t+1\right)}|{|}^{2}+\frac{\rho }{2},\;{\tilde{B}}^{t}=-Z_{3}^{t}-\rho V_{3}^{t}. \end{array}$$

To solve the subproblem given in Equation (31), the Lagrangian multiplier method is used, and the obtained update rule of U can be expressed as Equation (33):

(33) $$\begin{array}{c} U^{\left(t+1\right)}=\frac{{\tilde{Q}}^{t}-{\tilde{B}}^{t}}{2{\tilde{A}}^{t}}, \end{array}$$

where ${\tilde{Q}}^{t}=\left(1+\sum_{k=1}^{K}\frac{{\tilde{B}}^{t}}{2{\tilde{A}}^{t}}\right)/\sum_{k=1}^{K}\frac{1}{2{\tilde{A}}^{t}}$.

**$V_{3}$**-update: The subproblem with respect to $V_{3}$ is shown by Equation (34):

(34) $$\begin{array}{cc} V_{3}^{\left(t+1\right)} & =\underset{V_{3}}{\arg\min}\frac{\rho }{2}||V_{3}^{t}-U^{\left(t+1\right)}+\zeta_{3}^{t}|{|}_{F}^{2}+\frac{\rho }{2}||V_{3}^{t}H-V_{4}^{t}+\zeta_{4}^{t}|{|}_{F}^{2}. \end{array}$$

By setting the derivative of the objective function in Equation (34) to zero [32], the solution of $V_{3}$ is obtained as

(35) $$\begin{array}{c} V_{3}^{\left(t+1\right)}=\left(U^{\left(t+1\right)}-\zeta_{3}^{t}+V_{4}^{t}H^{T}-\zeta_{4}^{t}H^{T}\right){\left(I+HH^{T}\right)}^{-1}, \end{array}$$

where $Z_{3}=\zeta_{3}/\rho$ and $Z_{4}=\zeta_{4}/\rho$.

**$V_{4}$**-update: A suboptimization problem with respect to $V_{4}$ is written as

(36) $$\begin{array}{cc} V_{4}^{\left(t+1\right)} & =\underset{V_{4}}{\arg\min}\beta ||V_{4}^{t}|{|}_{1,1}+\frac{\rho }{2}||V_{4}^{t}-V_{3}^{\left(t+1\right)}H+\zeta_{4}^{t}|{|}_{F}^{2}. \end{array}$$

The soft threshold [32] is employed to update $V_{4}$, and the update rule of $V_{4}$ is given as

(37) $$\begin{array}{c} V_{4}^{\left(t+1\right)}=\mathrm{soft}\left(\beta /\rho ,V_{3}^{\left(t+1\right)}H-\zeta_{4}^{t}\right). \end{array}$$

Based on the obtained updating rules for variables A, S, U, C, $V_{1}$, $V_{2}$, $V_{3}$, $V_{4}$, $V_{5}$, $V_{6}$, and $V_{7}$, the detailed algorithm steps of our proposed CFNR method are summarized in Algorithm 1.
**Algorithm 1:** CFNR for Hyperspectral Band Selection
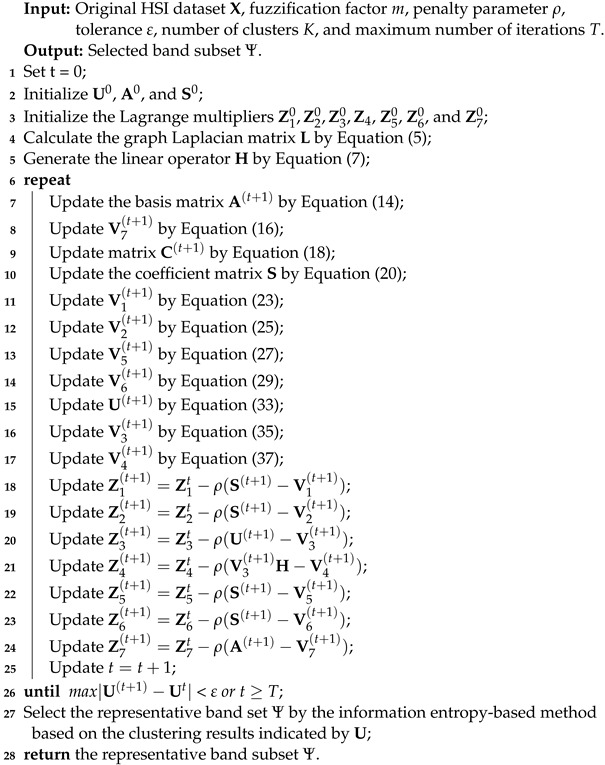


### 3.4. Information Entropy-Based Method for Representative Band Selection

After dividing all bands into different clusters, the next task is to select a group of representative bands from the obtained clusters. While most current band selection methods rely on selecting the band closest to its cluster centroid regarding Euclidean distance [12,37,38], this may be ineffective when dealing with noisy bands. In CFNR, considering the sensitivity of FCM to noise [39,40], we aim to select the target band subset via a method that can effectively reduce the effect of noise on the band selection. Specifically, to obtain the target band subset based on the clustering results, the proposed CFNR approach adopts the information entropy-based method [41,42], using which the band that contains the maximum amount of information in each cluster is selected as the representative band. This method is based on the assumption that bands should be selected based on the amount of information contained in the band. Specifically, the information entropy of all bands in each cluster is calculated and then sorted in descending order. Subsequently, the first band in each cluster is selected as the representative band. In this study, the information entropy $H(X_{:,l})$ of the band $X_{:,l}$, l=1,2,…,L, is calculated by

(38) $$\begin{array}{c} H\left(X_{:,l}\right)=-\sum_{\omega \in \Theta }p\left(\omega \right)logp\left(\omega \right), \end{array}$$

where ω denotes a grayscale value; Θ denotes a gray space, which contains all grayscale values of band $X_{:,l}$; and p(ω) denotes the probability distribution of ω in band $X_{:,l}$, which can be calculated using a grayscale histogram. Notably, the effects of noise interference on band selection can be avoided to a certain extent using the information entropy-based method [39].

## 4. Results and Discussion

### 4.1. Datasets

**Figure 2:**
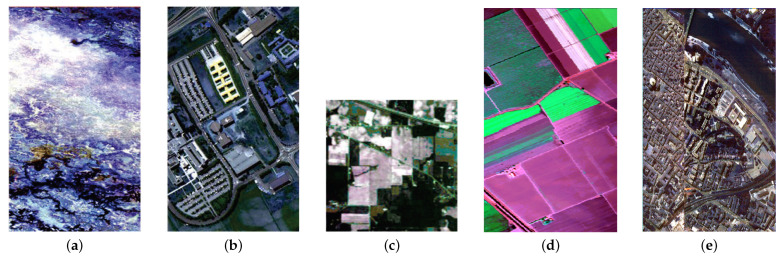
Images of five HSI datasets. (**a**) Botswana. (**b**) Pavia University. (**c**) Indian Pines. (**d**) Salinas. (**e**) Pavia Centre.

The five HSI datasets used in the experiments are concisely described. Table 1 and Figure 2 show the main information and images of these datasets, respectively.

The Botswana dataset comprises data obtained by the NASA EO-1 satellite over the Okavango Delta, Botswana in 2001. The size of the images in this dataset is 1476 × 256 pixels with a spatial resolution of 30 m. A total of 145 bands were retained in the experiments after removing noisy and uncalibrated bands with numbers (10–15, 82–97, 102–119, 134–164, and 187–220). This dataset has 14 identified classes.

The Pavia University dataset was acquired by the ROSIS sensor over the University of Pavia in Italy. The size of the images in this dataset is 610 × 340 pixels with a spatial resolution of 1.3 m, 115 spectral bands, and 16 classes. A total of 103 bands were retained in the experiments by excluding 12 noisy bands.

Indian Pines, one of the widely used test datasets for hyperspectral classification, comprises data obtained using the AVIRIS sensor in 1992. This dataset comprises images with a size of 145 × 145 pixels and a spatial resolution of 20 m with 220 bands in the spectral range of 400–2500 nm. The bands with numbers (104–108, 150–163, and 220) were removed to reduce the influence of water absorption bands, and the 200 retained bands were used in the experiments. In addition, this dataset includes 16 ground truth classes.

The Salinas dataset comprises data obtained by the AVIRIS sensor over the Salinas Valley in California, USA. The size of images in the Salinas dataset is 512 × 217 pixels with a spatial resolution of 3.7 m. A total of 204 bands were retained in the experiments after removing noisy and uncalibrated bands (108–112, 154–167, and 224). This dataset contains 16 identified classes.

The Pavia Centre dataset was acquired by the ROSIS sensor over the University of Pavia in Italy. This dataset contains 115 bands, excluding some bands that do not contain information, and 103 bands are retained in the experiment. The dataset has 9 classes of ground cover, and an image size of 1096 × 715 pixels.

### 4.2. Compared Methods

The proposed CFNR is compared with five representative band selection methods to evaluate its performance. These band selection methods are briefly introduced as follows.

1. MVPCA [10]: MVPCA is a representative ranking-based band selection method that first constructs a loading factor matrix from an eigenform matrix. All bands are then ranked in accordance with the loading factor matrix, and the top-ranked bands are ultimately selected as the representative band subset.

2. WaLuDi [15]: According to the correlation measure between bands based on Kullback–Leibler divergence, WaLuDi uses a hierarchical clustering algorithm based on Ward’s link method to continuously reduce the number of bands within a cluster continuously until an ideal subset of bands is obtained.

3. E-FDPC [16]: As a clustering-based band selection method, E-FDPC divides bands into clusters and calculates the score for each band by weighting the local density and intracluster distance. Based on the obtained scores, E-FDPC selects the bands with high scores as representative bands. In addition, E-FDPC can automatically determine the optimal number of representative bands through the introduction of isolated-point-stopping criterion.

4. ASPS [9]: ASPS is a clustering-based band selection method that first roughly divides the HSI to obtain a limited number of equal-sized subcubes. Subsequently, ASPS adjusts the subcubes by using the intercluster to intracluster distance ratio to obtain the subcubes with low correlation. Finally, ASPS selects the band with the least noise from each subcube as a representative band to form the target band subset.

5. HLFC [17]: HLFC is also a clustering-based band selection method. HLFC separates an HSI into multiple regions and then learns the corresponding low-dimensional latent features of each region through a superpixel segmentation algorithm. Subsequently, all the latent features of regions are integrated into a unified feature representation of the HSI. Finally, HLFC performs K-means clustering on the unified feature representation; the representative bands are selected from the obtained clusters using the information entropy-based method.

### 4.3. Experimental Setup

Two classifiers, namely linear discriminant analysis (LDA) [43] and support vector machine (SVM) [44], which adopts the radial basis function as the kernel function, are employed in the experiments to test the performance of the proposed methods. The proposed method is then compared with five representative band selection methods, comprising the clustering-based methods WaLuDi, E-FDPC, ASPS, HLFC, and the classical ranking-based method MVPCA. All the methods were implemented using MATLAB 2016b and executed on a computer using the Windows 10 operating system and Intel Core i7-9700K 3.60 GHz CPU.

The number of selected bands *K* in the experiments ranges from 5 to 50 with an interval of 5. Each experiment is performed 10 times, and the average results are reported. In CFNR, the matrices U are randomly initialized in [0, 1] and A and S are initialized using K-means [45]. Twenty percent of the samples are randomly selected for training and the rest are used for prediction. The values of tolerance ε, maximum number of iterations *T*, and penalty parameter ρ are set to $10^{-5}$, 200, and $10^{3}$, respectively. Each of the Lagrange multipliers $Z_{1}$, $Z_{2}$, $Z_{3}$, $Z_{4}$, $Z_{5}$, $Z_{6}$, and $Z_{7}$ is initialized as an all-ones matrix. Moreover, the model has four hyperparameters represented by three regularization parameters α, λ, and β and the dimension *P* of non-negative representation. The empirical values of these regularization parameters are shown in Table 2. For the dimension value *P* of non-negative representation, a *P* value that is substantially large results in a loss of explanation ability for low-dimensional representation learning, decreasing its benefits in band selection. Conversely, when *P* is set to a substantially small value, this may lead to substantial information loss. Therefore, the value of *P* is empirically set to 40. In addition, Table 3, Table 4, Table 5, Table 6 and Table 7 show the number of training and testing samples for each class in the five datasets. The experimental performance is evaluated using three criteria: overall accuracy (OA), average overall accuracy (AOA), and Kappa coefficient (Kappa).

### 4.4. Experimental Results

In this section, the results of a series of experiments conducted on five real datasets to demonstrate the effectiveness of our proposed CFNR method are presented.

#### 4.4.1. Classification Performance Comparison

**Figure 3:**
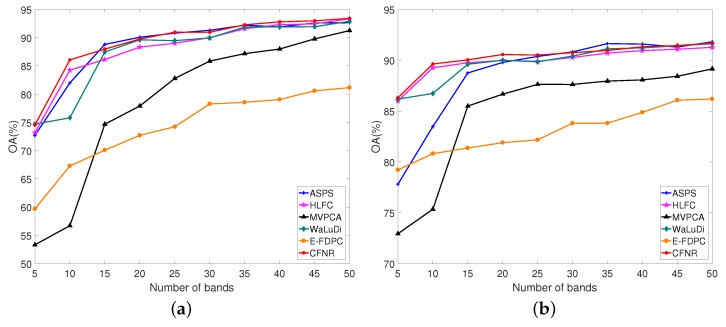
OA for the SVM and LDA classifiers by selecting different numbers of bands on the Botswana dataset. (**a**) OA by SVM. (**b**) OA by LDA.

**Figure 4:**
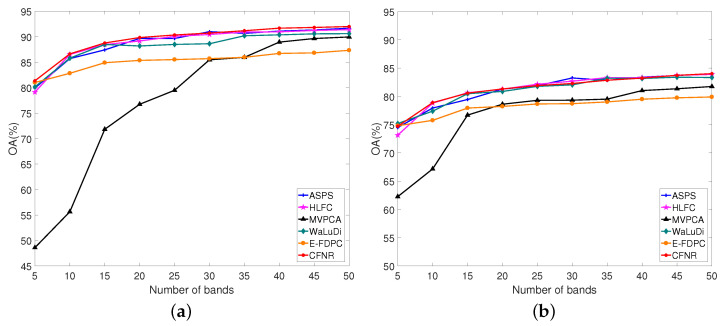
OA for the SVM and LDA classifiers by selecting different numbers of bands on the Pavia University dataset. (**a**) OA by SVM. (**b**) OA by LDA.

**Figure 5:**
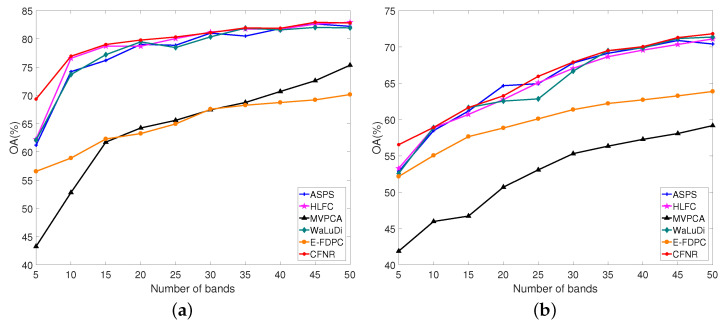
OA for the SVM and LDA classifiers by selecting different numbers of bands on the Indian Pines dataset. (**a**) OA by SVM. (**b**) OA by LDA.

**Figure 6:**
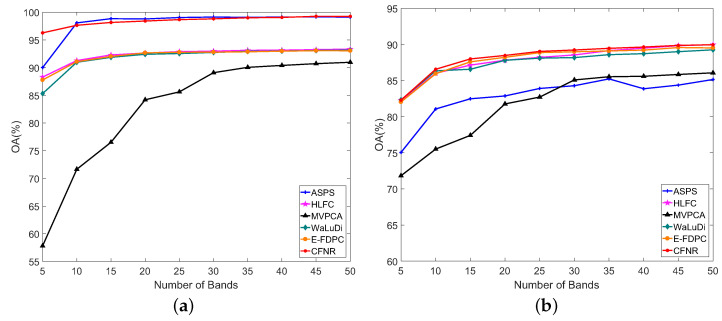
OA for the SVM and LDA classifiers by selecting different numbers of bands on the Salinas dataset. (**a**) OA by SVM. (**b**) OA by LDA.

**Figure 7:**
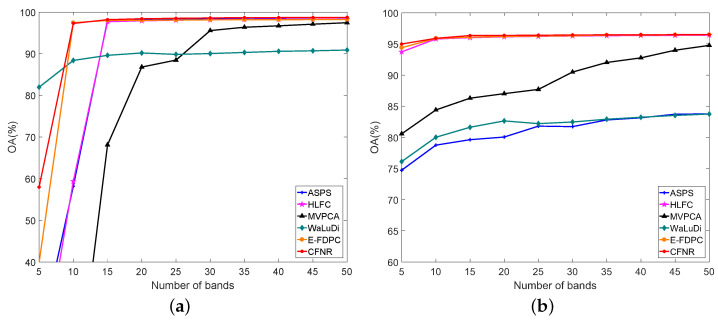
OA for the SVM and LDA classifiers by selecting different numbers of bands on the Pavia Centre dataset. (**a**) OA by SVM. (**b**) OA by LDA.

Table 8 shows the values of AOA and Kappa obtained using different methods on the five datasets, where AOA and Kappa represent the average performance over the number of bands ranging from 5 to 50 with an interval of 5. In Table 8, the columns represent the dataset, classifier, and names of the methods and the rows represent the classification accuracy of the dataset with different methods. The values in red font represent the best results. As shown in Table 8, the classification performance of the proposed CFNR outperforms that of other methods on five datasets. The performance of HLFC was the second best when using the SVM classifier on the Pavia University and Indian Pines datasets. When using the LDA classifier, the Kappa of HLFC is the same as our proposed method on the Pavia University dataset. MVPCA performs poorly on all five datasets when using the SVM classifier, with CFNR, HLFC, ASPS, and WaLuDi providing better performance. For example, for the Botswana dataset, HLFC and ASPS exhibit good performance when using the SVM, but CFNR yields superior results. CFNR demonstrates a better AOA than MVPCA, WaLuDi, DPC, ASPS, and HLFC by 10.40%, 1.58%, 14.98%, 0.09%, and 1.06%, respectively. Similar performance is demonstrated in the case of the other four datasets when using the SVM classifier. The advantage of CFNR on the Indian Pines and Pavia University datasets is not evident compared with HLFC and ASPS in the LDA classifier. However, CFNR still achieves good results in the LDA classifier. Overall, the effectiveness of the CFNR method is demonstrated by comparing its AOA and Kappa with those of other methods. In addition, Figure 3, Figure 4, Figure 5, Figure 6 and Figure 7 shows the curves of the OA values, based on which all six band selection methods are compared when using SVM and LDA classifiers on the five datasets.

(1) Botswana dataset: Figure 3a,b shows the results of using the SVM and LDA classifiers on the Botswana dataset. According to Figure 3a, CFNR provides satisfactory performance for most of the selected bands. For example, CFNR achieves excellent performance when 10, 40, and 45 bands are selected. When 25 and 35 bands are selected, the OA values of CFNR are similar to those of ASPS, but remain higher than those of the other methods. CFNR demonstrates the second-best performance when the number of selected bands is 15, 20, and 30. In particular, in the case of 50 selected bands, the performance of CFNR is similar to that of HLFC, while surpassing those of the WaLuDi, ASPS, MVPCA, and E-FDPC. HLFC demonstrates the second-best performance when the number of selected bands is 10. The performance of CFNR is considerably better than that of HLFC when 50 bands are selected. Although the performance of CFNR is similar to that of WaLuDi when 5 bands are selected, CFNR performs better than HLFC, ASPS, MVPCA, and E-FDPC. Furthermore, CFNR exhibits excellent performance when using the LDA classifier. As shown in Figure 3b, CNFR exhibits the best performance when the number of selected bands ranges from 5 to 25. Although CFNR performs slightly worse than ASPS when 30, 40, and 50 bands are selected, CFNR still outperforms HLFC, WaLuDi, and E-FDPC. When 45 bands are selected, the performance of CFNR is similar to that of WaLuDi but is still better than that of HLFC, E-FDPC, and MVPCA.

(2) Pavia University dataset: Figure 4a,b verifies the performance of CFNR on the Pavia University dataset. As shown in Figure 4a, CFNR achieves superior results when using the SVM classifier. For example, except when 10 and 30 bands are selected, the proposed method achieves excellent performance. Although the OA of CFNR is inferior to ASPS when 30 bands are selected, it still outperforms the other methods. At 10 bands, the performance of CFNR is similar to that of HLFC and better than those of WaLuDi, ASPS, E-FDPC, and MVPCA. When 10 and 15 bands are selected, the performance of CFNR is similar to that of HLFC. In the rest of the cases, the performances of CFNR exceed those of HLFC. Considering the results of the LDA classifier shown in Figure 4b, the performance of CFNR is not inferior to the other methods. Specifically, in cases of selecting 20, 45, and 50 bands, the performances of CFNR are similar to those of HLFC and ASPS and superior to those of WaLuDi, MVPCA, and E-FDPC. When 10 bands are selected, CFNR performs similarly to HLFC, and it outperforms ASPS, WaLuDi, E-FDPC, and MVPCA. When 30 bands are selected, the performance of CFNR is superior to those of WaLuDi, MVPCA, and E-FDPC. When 30 bands are selected, HLFC achieves the second-best performance and is better than CFNR. Furthermore, the OA values of the CFNR, ASPS, WaLuDi, and HFLC are similar when 40 bands are selected, but CFNR exhibits superior performance than the other methods.

(3) Indian Pines dataset: Similarly, for the Indian Pines dataset, Figure 5a,b shows that our proposed method exhibits outstanding performance compared with that of the other methods. In particular, CFNR has a distinct advantage in experiments conducted on the SVM classifier. As shown in Figure 5a, our proposed method works best on almost all bands in the SVM classifier. CFNR achieves satisfactory classification performance when the numbers of selected bands are 5–25 and 45. When 30 and 50 bands are selected, the performance of CFNR is similar to that of HLFC and better than that of WaLuDi, MVPCA, and E-FDPC. In other cases, CFNR performs no worse than the other methods. According to Figure 5b, the performance of CFNR is superior for most of the selected bands when using the LDA classifier. At 5, 25, 45, and 50 bands, CFNR demonstrates excellent performance. CFNR achieves the second-best performance with 20 selected bands. Moreover, when the number of selected bands is 15 and 35, the performance of CFNR is similar to that of WaLuDi and superior to the other methods. When 5 bands are selected, HLFC demonstrates the second-best performance. At 10 bands, the performances of CFNR, WaLuDi, and HLFC are similar and superior to those of ASPS, E-FDPC, and MVPCA.

(4) Salinas dataset: Figure 6a,b shows the results for the SVM and LDA classifiers on the Salinas dataset. According to Figure 6a, CFNR outperforms most methods when using the SVM classifier. Specifically, CFNR demonstrates excellent performance when 5 bands are selected. For 10–30 selected bands, the OA values of CFNR are the second best. For 35–50 selected bands, the performance of CFNR is similar to that of ASPS and surpasses those of WaLuDi, HLFC, MVPCA, and E-FDPC. In addition, the advantage of CFNR is more apparent when using the LDA classifier, as shown in Figure 6b. In Figure 6b, the OA of CFNR is the best when the number of selected bands ranges from 10 to 35. CFNR performs similarly to HLFC when the number of selected bands ranges from 40 to 50 and still outperforms ASPS, WaLuDi, and E-FDPC. When 5 bands are selected, the performance of CFNR is similar to that of WaLuDi, E-FDPC, and HLFC but still better than those of ASPS and MVPCA.

(5) Pavia Centre dataset: Figure 7a,b verifies the performance of CFNR on the Pavia Centre dataset. CFNR exhibits good performance for the SVM classifier in Figure 7a. For example, when the number of selected bands is 5, the proposed CFNR method achieves the second-best performance, but its performance is better than those of MVPCA, E-FDPC, ASPS, and HLFC. At 10 bands, the OA of CFNR is similar to that of E-FDPC and better than those of MVPCA, WaLuDi, E-FDPC, ASPS, and HLFC. When the numbers of selected bands range from 20 to 50, CFNR has a slight advantage over the other methods. Considering the results of the LDA classifier shown in Figure 7b, the performance of CFNR is not inferior to those of the other methods. In particular, CFNR performs best when the number of selected bands is 5, 15, and 20. When 25–50 bands are selected, the performances of CFNR are similar to those of HLFC and ASPS but superior to those of the E-FDPC, MVPCA, and WaLuDi.

**Figure 8:**
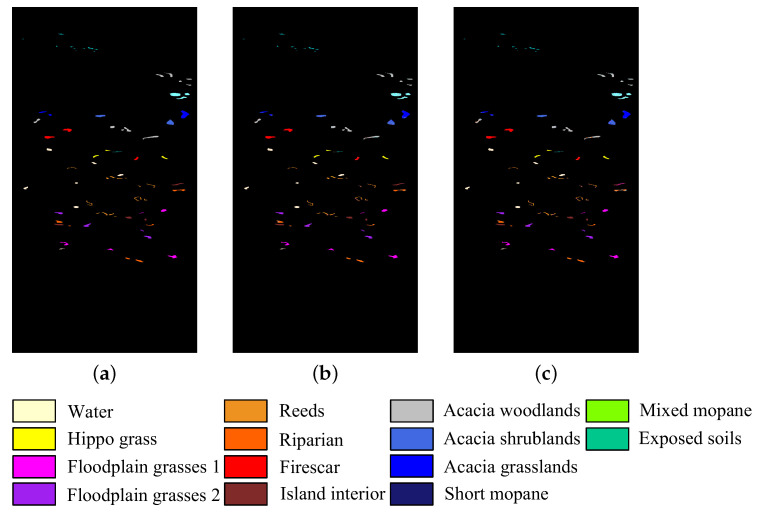
Classification map of CFNR on Botswana dataset when the band is 30. (**a**) Ground truth. (**b**) SVM. (**c**) LDA.

**Figure 9:**
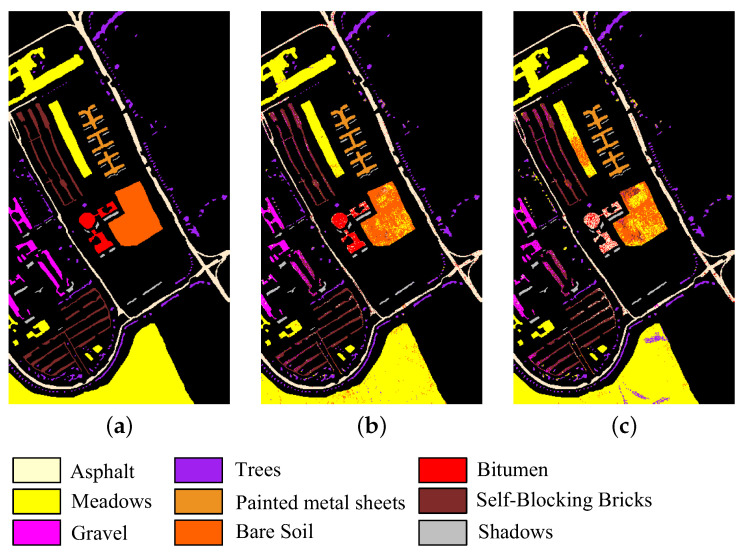
Classification map of CFNR on Pavia University dataset when the band number is 30. (**a**) Ground truth. (**b**) SVM. (**c**) LDA.

**Figure 10:**
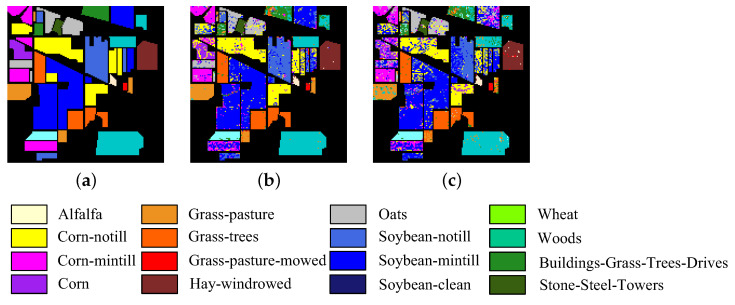
Classification map of CFNR in Indian Pine dataset when the band number is 30. (**a**) Ground truth. (**b**) SVM. (**c**) LDA.

**Figure 11:**
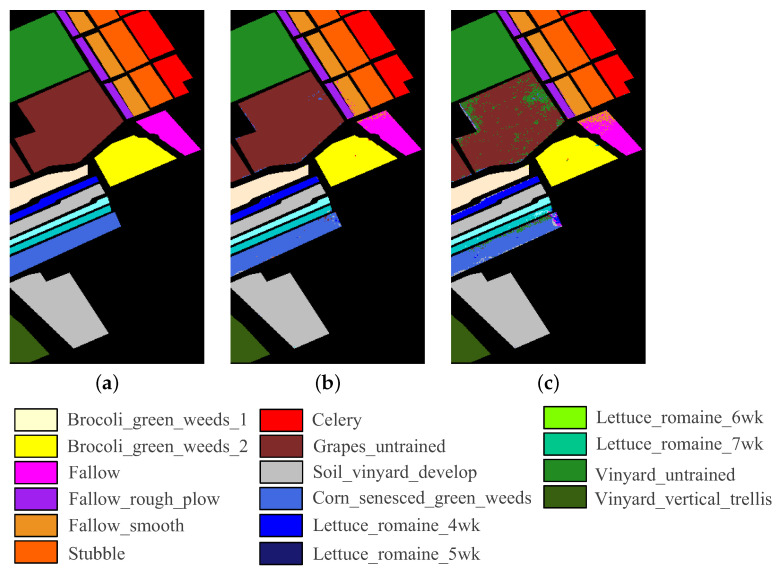
Classification map of CFNR on Salinas dataset when the band number is 30. (**a**) Ground truth. (**b**) SVM. (**c**) LDA.

**Figure 12:**
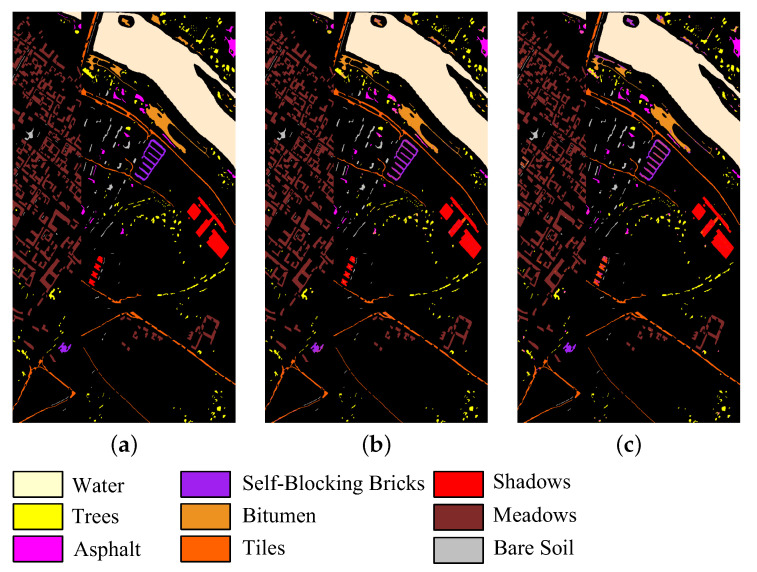
Classification map of CFNR on Pavia Centre dataset when the band number is 30. (**a**) Ground truth. (**b**) SVM. (**c**) LDA.

In addition, to provide an intuitive description of the quality of the bands selected by CFNR, Figure 8, Figure 9, Figure 10, Figure 11 and Figure 12 display the classification maps afforded by SVM and LDA when CFNR is used to select 30 bands on each of the 5 datasets. By comparing the ground truth and classification maps afforded by SVM and LDA as shown in Figure 8, Figure 9, Figure 10, Figure 11 and Figure 12, we can see that CFNR evidently demonstrates satisfactory results under the condition of removing 79%, 85%, 70%, 85%, and 70% of the bands in the Botswana, Indian Pines, Pavia University, Salinas, and Pavia Centre datasets, respectively.

Overall, our proposed CFNR method shows satisfactory results for the five datasets. According to the experimental results, the performance of CFNR is outstanding when using the SVM classifier. In addition, although the effect of CFNR is not as good as that of SVM on the LDA classifier, it is superior to those of other comparison methods in many cases. Therefore, CFNR provides a good classification and can select a band subset that meets the requirements of hyperspectral classification applications, verifying the effectiveness of our method.

#### 4.4.2. Convergence Analysis

**Figure 13:**
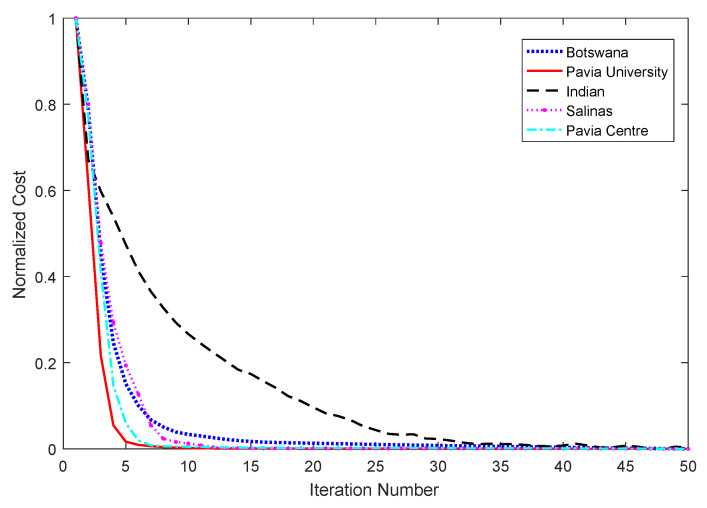
Convergence curves of our proposed method on the five datasets.

Figure 13 shows the plotted convergence curve of CFNR for five datasets for the case of 30 selected bands to demonstrate the convergence of the CFNR method. This figure reveals that the algorithm of CFNR is looped 50 times for each of the 5 datasets, in which the normalized cost of the objective function of CFNR is used. The algorithm converges after 15 loops on the Indian Pines dataset. On the Pavia University and Botswana datasets, the proposed method converges approximately around 35 and 5 loops, respectively. When CFNR is tested on the Salinas and the Pavia Centre datasets, it converges after 7 and 9 loops, respectively.

### 4.5. Experimental Discussion

The findings from our experiments as well as the advantages and limitations of our method are presented in this section; moreover, suggestions for future work are presented.

1. Principal findings and comparison with other studies. CFNR is compared with ranking and clustering-based band selection methods. The experimental results in Figure 3, Figure 4, Figure 5, Figure 6 and Figure 7 reveal that the ranking and clustering-based methods exhibit relatively poor performance when the number of selected bands is small. This finding implies that it is considerably difficult to use fewer bands and provide sufficient information. In addition, as shown in Table 8 and Figure 3, Figure 4, Figure 5, Figure 6 and Figure 7, most clustering-based methods outperform ranking-based methods. The presumed reason is that the ranking-based band selection methods are typically based on a single criterion. These findings are consistent with those of previous studies [46,47]. Moreover, as shown in Figure 3, Figure 4, Figure 5, Figure 6 and Figure 7, the OA values of all methods increase with the increasing number of bands, but the rate of increase becomes progressively slow. This is because as the number of selected bands increases, more feature information is included in the representative bands, thereby increasing redundancy [48].

2. Advantages of the proposed method. According to the experiments shown in Figure 3, Figure 4, Figure 5, Figure 6 and Figure 7 and Table 8, the main advantages of CFNR lie in its superior performance in the SVM classifier and its robustness against datasets. As shown in Figure 3, Figure 4, Figure 5, Figure 6 and Figure 7, compared with other clustering-based band selection methods, the proposed CFNR shows good performance for all 5 datasets when the numbers of selected bands are 5–10 and 35–50 in the SVM classifier. On the one hand, the non-negative expression based on manifold learning in CFNR successfully finds low-dimensional discriminative representations for clustering the HSIs. On the other hand, the constrained FCM model in CFNR provides improved clustering results required for band selection tasks. Table 8 shows that most methods perform substantially worse on the Indian dataset than the four other datasets, which may be due to important feature information being contained in the removed noise bands of the Indian dataset [9]. However, the CFNR method still demonstrates better performance than the other methods on the Indian dataset when using SVM. This finding indicates that the bands selected by our proposed method are highly discriminative for the SVM classifier.

3. Limitations of the study. One limitation of our proposed approach lies in the presence of four hyperparameters, introducing some inconvenience to the applications of the proposed CFNR method. Nevertheless, the excellent performance of the proposed method addresses this limitation. Furthermore, the influence of noise is considered to a certain extent using the information entropy-based method in CFNR. However, how noise is handled merits further consideration. Determining the hyperparameter values through an adaptive solution and removing noise in representation learning should also be emphasized in future research.

## 5. Conclusions

A novel hyperspectral band selection method named CFNR is introduced in this study. In the proposed method, GNMF is integrated into the FCM model, by which clustering can be performed on the discriminative non-negative representation of all bands of a target HSI. Specifically, by exploiting the intrinsic manifold structure of HSIs with the help of GNMF, the discriminative non-negative representation of each band is determined. A correlation constraint is imposed on the membership matrix in the model of the proposed method to exploit the band correlation property of HSIs. Consequently, the similarity of clustering assignments among neighboring bands is enforced. This condition is favorable for obtaining clustering results that are consistent with the requirements of band selection. In addition, the proposed approach adopts the information entropy-based method to select a representative band subset from the obtained clusters. Compared with existing clustering-based band selection methods, CFNR designs an effective joint learning model of clustering and representative learning for band selection. As a result, clustering can be performed on the discriminative non-negative representation of all bands rather than on the original high-dimensional hyperspectral bands. Additionally, ADMM is used to provide an optimized solution for the proposed CFNR model. Various experiments on the Indian Pines, Botswana, Pavia University, Salinas, and Pavia Centre datasets indicate that the proposed method can provide superior performance compared with several state-of-the-art methods.

## Figures and Tables

**Table 1:** Information of the five HSI datasets.

Dataset	Pixels	Spatial Resolution	Classes	Bands
Botswana	1476×256	30 m/pixel	14	145
Pavia University	610×340	1.3 m/pixel	9	103
Indian Pines	145×145	20 m/pixel	16	200
Salinas	512×217	3.7 m/pixel	16	204
Pavia Centre	1096×715	1.3 m/pixel	9	102

**Table 2:** Hyperparameters settings for the five datasets.

Dataset	α	λ	β
Botswana	10	1.2	0.02
Pavia University	12	1.1	0.03
Indian Pines	12	1.2	0.01
Salinas	11	1.2	0.02
Pavia Centre	12	1.1	0.01

**Table 3:** Number of training and testing samples in the Botswana dataset.

Class	Name	Training Samples	Testing Samples
1	Water	54	216
2	Hippo grass	20	81
3	Floodplain grasses 1	50	201
4	Floodplain grasses 2	43	172
5	Reeds	54	215
6	Riparan	54	215
7	Firescar	52	207
8	Island interior	41	162
9	Acacia woodlands	63	251
10	Acacia shrublands	50	198
11	Acacia grasslands	61	244
12	Short mopane	36	145
13	Mixed mopane	54	214
14	Exposed soils	19	76

**Table 4:** Number of training and testing samples in the Pavia University dataset.

Class	Name	Training Samples	Testing Samples
1	Asphalt	1326	5305
2	Meadows	3730	14,919
3	Gravel	420	1679
4	Trees	613	2451
5	Painted metal sheets	269	1076
6	Bare Soil	1006	4023
7	Bitumen	266	1064
8	Self-Blocking Bricks	736	2946
9	Shadows	1894	7576

**Table 5:** Number of training and testing samples in the Indian Pine dataset.

Class	Name	Training Samples	Testing Samples
1	Alfalfa	9	37
2	Corn-notill	286	1142
3	Corn-mintill	166	664
4	Corn	47	190
5	Grass-pasture	97	386
6	Grass-trees	146	584
7	Grass-pasture-mowed	6	22
8	Hay-windrowed	96	382
9	Oats	4	16
10	Soybean-notill	194	778
11	Soybean-mintill	491	1964
12	Soybean-clean	119	474
13	Wheat	41	164
14	Woods	253	1012
15	Buildings-Grass-Trees-Drives	77	309
16	Stone-Steel-Towers	19	74

**Table 6:** Number of training and testing samples in the Salinas dataset.

Class	Name	Training Samples	Testing Samples
1	Brocoli_green_weed_1	402	2009
2	Brocoli_green_weeds_2	745	3726
3	Fallow	395	1976
4	Fallow_rough_plow	279	1394
5	Fallow_smooth	536	2678
6	Stubble	792	3959
7	Celery	716	3579
8	Grapes_untrained	2254	11,271
9	Soil_vinyard_develop	1241	6203
10	Corn_senesced_green_weeds	656	3278
11	Lettuce_romaine_4wk	214	1068
12	Lettuce_romaine_5wk	385	1927
13	Lettuce_romaine_6wk	16	916
14	Lettuce_romaine_7wk	203	1017
15	Vinyard_untrained	1454	7268
16	Vinyard_vertical_trellis	361	1807

**Table 7:** Number of training and testing samples in the Pavia Centre dataset.

Class	Name	Training Samples	Testing Samples
1	Water	168	842
2	Trees	164	820
3	Asphalt	163	816
4	Self-Blocking Bricks	162	808
5	Bitumen	162	808
6	Tiles	252	1260
7	Shadows	95	476
8	Meadows	16	824
9	Bare Soil	164	820

**Table 8:** Performance comparison on the five datasets, where the values in red bold font represent the best results. CFNR is our proposed method.

Dataset	Classifier	CFNR	MVPCA [10]	WaLuDi [15]	E-FDPC [16]	ASPS [9]	HLFC [17]
Botswana	AOA(SVM)	**89.143**	78.744	87.561	74.160	89.050	88.081
	Kappa(SVM)	**0.8824**	0.7695	0.8652	0.7200	0.8813	0.8709
	AOA(LDA)	**90.324**	84.930	89.923	83.022	89.160	89.917
	Kappa(LDA)	**0.8968**	0.8406	0.8926	0.8204	0.8847	0.8925
Pavia University	AOA(SVM)	**89.423**	77.230	88.150	85.219	88.820	88.834
	Kappa(SVM)	**0.8582**	0.6661	0.8409	0.7999	0.8497	0.8499
	AOA(LDA)	**81.372**	76.708	81.129	78.232	81.230	81.359
	Kappa(LDA)	**0.7685**	0.7120	0.7656	0.7308	0.7679	**0.7685**
Indian Pines	AOA(SVM)	**79.451**	64.231	77.893	64.971	77.812	78.556
	Kappa(SVM)	**0.7634**	0.5805	0.7450	0.5888	0.7435	0.7534
	AOA(LDA)	**65.569**	52.446	64.788	59.720	65.460	64.698
	Kappa(LDA)	**0.6314**	0.4913	0.6236	0.5722	0.6304	0.6225
Salinas	AOA(SVM)	**98.465**	82.713	91.812	92.111	98.049	92.333
	Kappa(SVM)	**0.9826**	0.8055	0.9086	0.9120	0.9767	0.9145
	AOA(LDA)	**88.257**	81.738	87.493	87.908	82.830	87.845
	Kappa(LDA)	**0.8725**	0.8030	0.8644	0.8688	0.8143	0.8682
Pavia Centre	AOA(SVM)	**94.019**	73.041	89.250	92.222	86.631	85.5632
	Kappa(SVM)	**0.9309**	0.7027	0.8562	0.9132	0.9573	0.8461
	AOA(LDA)	**96.233**	89.001	81.849	96.091	81.014	95.961
	Kappa(LDA)	**0.9471**	0.8478	0.7742	0.9451	0.7644	0.9433

## Data Availability

Not applicable.

## Abbreviations

The following abbreviations are used in this manuscript:

HSIs	Hyperspectral remote sensing images
MVPCA	Maximum-variance principal component analysis
WaLuDi	Ward’s linkage strategy using divergence
E-FDPC	Enhanced fast density-peak-based clustering
ASPS	Adaptive subspace partition strategy
HLFC	Region-aware hierarchical latent feature representation learning-guided clustering
NMF	Non-negative matrix factorization
GNMF	Graph regularized non-negative matrix factorization
FCM	Fuzzy C-means
ADMM	Alternating direction multiplier method
SVM	Support vector machine
LDA	Linear discriminant analysis
OA	Overall accuracy
AOA	Average overall accuracy
CFNR	Joint learning of correlation constrained fuzzy clustering and discriminative non-negative representation
